# Impact of Non-steroidal Anti-inflammatory Drug Administration for 12 Months on Renal Function

**DOI:** 10.3389/fpain.2021.644391

**Published:** 2021-05-26

**Authors:** Kazuhiro Hayashi, Kenji Miki, Hiroshi Kajiyama, Tatsunori Ikemoto, Masao Yukioka

**Affiliations:** ^1^Multidisciplinary Pain Center, Aichi Medical University, Nagakute, Japan; ^2^Center for Pain Management, Hayaishi Hospital, Osaka, Japan; ^3^Faculty of Health Science, Osaka Yukioka College of Health Science, Osaka, Japan; ^4^Department of Rheumatology and Applied Immunology, Saitama Medical University, Saitama, Japan; ^5^Department of Orthopedic Surgery, Aichi Medical University, Nagakute, Japan

**Keywords:** anti-inflammatory agents, analgesics, drug-related side effects and adverse reactions, longitudinal studies, kidney, musculoskeletal pain

## Abstract

**Background:** The use of non-steroidal anti-inflammatory drugs (NSAIDs) is associated with an increased risk of renal complications. Resolution of renal adverse effects after NSAID administration has been observed after short-term use. Thus, the present study aimed to investigate a series of patients with chronic musculoskeletal pain who underwent long-term NSAID administration followed by switching to tramadol hydrochloride/acetaminophen (TA) combination tablets to study the impact of NSAID-induced renal adverse effects.

**Methods:** This was a longitudinal retrospective study of 99 patients with chronic musculoskeletal pain. The patients were administrated with NSAIDs daily during the first 12 months, followed by daily TA combination tablets for 12 months. Estimated glomerular filtration rate (eGFR) and serum levels of aspartate aminotransferase and alanine transaminase were measured at baseline, after NSAID administration and after TA administration.

**Results:** eGFR was significantly reduced after 12-month NSAID administration (median, from 84.0 to 72.8 ml/min/1.73 m^2^), and the reduction was not shown after the subsequent 12-month TA administration (median, 71.5 ml/min/1.73 m^2^). Reduction in eGFR was less in patients who received celecoxib (median, −1.8 ml/min/1.73 m^2^) during the first 12 months. There was no significant difference in aspartate aminotransferase and alanine transaminase in each period.

**Conclusions:** Thus, patients receiving NSAIDs for 12 months displayed both reversible and irreversible reduction of eGFR upon cessation of NSAIDs and switching to TA. Our data highlight the potential safety benefit of utilizing multimodal analgesic therapies to minimize the chronic administration of NSAIDs.

## Introduction

The administration of non-steroidal anti-inflammatory drugs (NSAIDs) to treat chronic musculoskeletal pain has become widely used in the clinic due to its ability to provide effective levels of pain relief [1–6]. However, regular administration of NSAIDs has an increased risk of gastrointestinal, cardiovascular, and renal complications [1–6]. There is a linear relationship between NSAID cumulative dose and change in renal function over a 2-year period [7]. Despite the high incidence of dose/duration-dependent renal adverse effects (estimated at 1–5%) [7, 8], there is a paucity of data regarding the long-term safety of NSAID therapy, and the risk of renal damage has prompted an increasing appreciation in the value of multimodal analgesia in the management of moderate-to-severe pain. For example, tramadol hydrochloride/acetaminophen (TA) combination tablets have emerged as a particularly useful option for chronic pain management [5, 6].

Previous studies have demonstrated that the renal adverse effects of NSAIDs are usually reversible [8–10], but such studies have several limitations. For example, Chou et al. showed the risk of kidney injury is higher in current NSAID users than in past NSAID users *vs*. control [9], which suggests the renal risks from NSAIDs could be reversible. However, they defined past NSAID users as having a termination date of 31–180 days before the index date, regardless of the administration period. Moreover, Shukla et al. reported that rises in kidney injury biomarkers resulting from regular NSAID therapy for spondyloarthritis are seen as early as 1 week and continue to rise up to 6 weeks [10]. Notably, the same study also showed reversibility in the rise of kidney injury biomarkers at 12 weeks upon stopping the drug [10]. Taken together, these studies show that regular administration of NSAIDs results in chronic renal failure [8], but patients taking NSAIDs for 6 weeks or less may have a chance of recovery [10]. Based on the potentially intolerable adverse effects or suboptimal pain relief, substantial proportions of musculoskeletal pain patients are often switched to a different treatment within 12 months of initiating NSAID treatment [11–13]. However, no study to date has evaluated the potential safety benefit of this common practice: reversing renal adverse effects after cessation of long-term NSAID therapy.

Thus, the present study aimed to investigate a series of patients with chronic musculoskeletal pain who underwent long-term NSAID administration followed by switching to TA combination tablets to study the impact of NSAID-induced renal adverse effects.

## Materials and Methods

### Subjects

**Figure 1:**
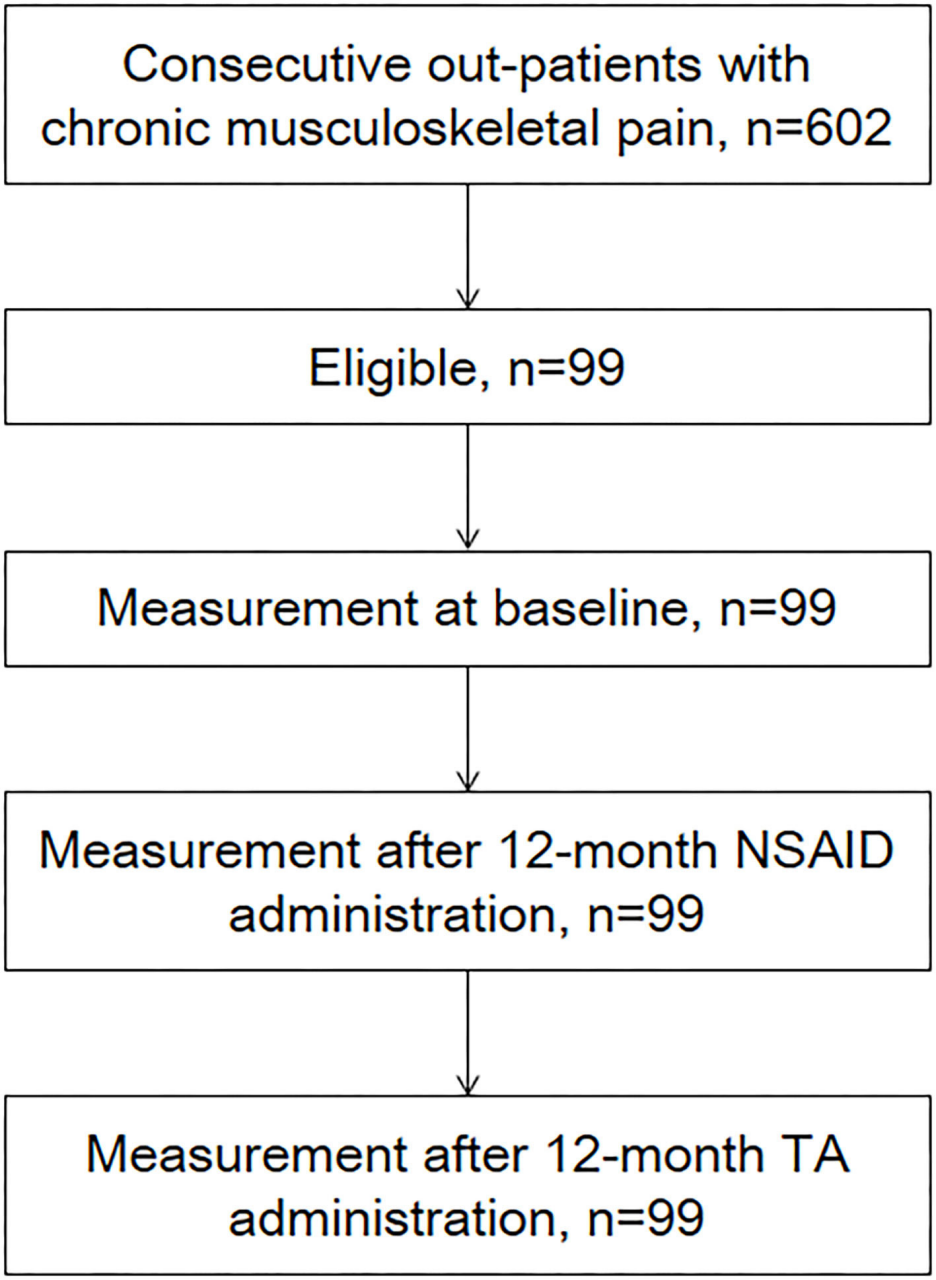
Flowchart of participants through the study. Ninety-nine patients were analyzed in this study.

The Research Ethics Committee of Amagasaki Central Hospital approved this study (no. H23022501). Data were retrospectively collected from medical records of 602 consecutive outpatients with chronic musculoskeletal pain from July 2011 to February 2012 at a primary care clinic. Inclusion criteria included age ≥ 20 years old, the existence of chronic musculoskeletal pain over the follow-up period of 2 years, and receiving daily NSAIDs during the first 12 months followed by receiving daily TA combination tablets for 12 months. Chronic musculoskeletal pain was defined as persisting, continuous, or intermittent pain for longer than 3 months [14]. Exclusion criteria were cancer-related pain, presence of neurological signs, evidence of bone fractures, recent surgery within the past 6 months, positive pregnancy test, American Society of Anesthesiologists' physical status ≥ 3, allergy or contraindication to the tested substances, severe kidney [estimated glomerular filtration rate (eGFR) < 30] or liver function disorders (Child–Pugh classes A, B, and C), acute duodenal or ventricular ulcer, or laboratory data outside of normal ranges. Finally, 99 patients receiving daily NSAIDs during the first 12 months followed by receiving daily TA combination tablets for 12 months were analyzed in this study (Figure 1). The patients were included regardless of administration dose. Concomitant medications were not permitted.

The number of subjects was determined by a sample size estimation using G^*^Power software (v 3.0.10; Franz Faul, Kiel University, Kiel, Germany). On the basis of the effect size of 0.3, the minimum number of subjects was estimated to be 90 for an α-level of 0.05 and a power (1–β) of 0.80.

### Treatment Characteristics

NSAIDs used in the study included meloxicam, loxoprofen, diclofenac, celecoxib, and others. During the latter 12 month period, all study participants were administrated daily TA combination tablets (Ultracet^®^). Change of administration dose was permitted. The initial dosage and administration of TA was one tablet (tramadol hydrochloride 37.5 mg and acetaminophen 325 mg) given orally four times per day [15]. The dose could be increased or decreased depending on patients' symptoms, but no more than two tablets per administration were permitted (up to a maximum of eight tablets daily). No other supplementary analgesic medications were given during the study. Discontinuation of medication for the treatment of internal comorbidities was not required.

### Outcomes

Patient characteristics included age, sex, major diagnosis, comorbidities, number of medications for comorbidities, and administration dose. Laboratory values were routinely collected at baseline, after 12-month NSAID administration and after 12-month TA administration. Comparisons of laboratory results during the 12 months with daily NSAIDs and during the following 12 months with daily administration of TA combination tablets were made in the same patient.

The primary outcome measure was serum levels of eGFR. eGFR was calculated as follows [16]: 194 × age-0.287 × serum creatinine-1.094 (if female, ×0.739). The eGFR values (ml/min/1.73 m^2^) in a given range were stratified into one of the following published chronic kidney disease (CKD) categories [17]: grade 1, normal or high, ≥90; grade 2, mildly decreased, 60–89; grade 3a, mildly to moderately decreased, 45–59; grade 3b, moderately to severely decreased, 30–44; grade 4, severely decreased, 15–29; grade 5, kidney failure, <15; or dialysis. Patients categorized with an increase in severity of at least one grade in the CKD category were enrolled for NSAID and TA administration.

Secondary outcome measures were serum levels of aspartate transaminase (AST) and alanine transaminase (ALT). Other information regarding adverse events during treatment was also collected. Treatment outcome measures were assessed at baseline and after treatment, in each treatment, by using a pain-Numeric Rating Scale (NRS) [18]. The pain-NRS was used to measure pain severity at each assessment, where 0 = no pain and 10 = worst pain imaginable [18].

### Statistical Analysis

Relative change in eGFR, AST, and ALT from baseline (xb) and measurements was calculated using the equation (x–xb)/xb, where x is the measured value. The normality of distribution for each measurement was evaluated using the Shapiro–Wilk test for continuous variables. The outcome variables were not normally distributed; thus, continuous data are expressed as medians and interquartile ranges (IQRs). Categorical variables were analyzed using the chi-square test. Continuous variables were analyzed using the Mann–Whitney *U*-test, the Kruskal–Wallis test, the Friedman test, the Steel–Dwass test, and the Spearman's rank correlation coefficient test.

All data were statistically analyzed using the SPSS 25.0J program, and *P* < 0.05 were considered significant.

## Results

Of the 99 patients, 70 (71%) were female (Table 1). The median age was 73 years (IQR, 47–81). Major diagnoses (multiple allowed) of the patients included lumbago (*n* = 45), osteoarthritis (*n* = 28), and rheumatoid arthritis (*n* = 3). NSAIDs taken during the first 12 months included meloxicam (*n* = 31), loxoprofen (*n* = 25), diclofenac (*n* = 13), and celecoxib (*n* = 20). No significant difference in patient characteristics, pain conditions, comorbidities, number of medications for comorbidities, and pain-NRS were observed based on the particular NSAIDs used (Tables 1, 2). No other serious and minor complications occurred during the 2-year research period.

**Table 1:** Patient characteristics.

	**Overall** **(*n* = 99)**	**Meloxicam** **(*n* = 31)**	**Loxoprofen** **(*n* = 25)**	**Diclofenac** **(*n* = 13)**	**Celecoxib** **(*n* = 20)**	**Other** **(*n* = 10)**
**Demographics**						
Age [year]	73 [47–81]	68 [45–81]	80 [59–83]	73 [45–82]	71 [47–80]	77 [68–83]
Female, n (%)	70 (71%)	23 (74%)	17 (68%)	8 (62%)	16 (80%)	6 (60%)
**Major diagnoses (multiple allowed)**						
Lumbago, n (%)	45 (45%)	11 (35%)	17 (68%)	5 (38%)	8 (40%)	4 (40%)
Osteoarthritis, n (%)	28 (28%)	7 (23%)	4 (16%)	4 (31%)	9 (45%)	4 (40%)
Rheumatoid arthritis, n (%)	3 (3%)	3 (10%)	0 (0%)	0 (0%)	0 (0%)	0 (0%)
**Comorbidities**						
Diabetes, n (%)	4 (4%)	0 (0%)	2 (8%)	1 (8%)	0 (0%)	1 (10%)
Hypertension, n (%)	22 (22%)	4 (13%)	9 (36%)	4 (31%)	3 (15%)	2 (20%)
Chronic heart failure, n (%)	3 (3%)	0 (0%)	2 (8%)	0 (0%)	1 (5%)	0 (0%)
Dyslipidemia, n (%)	1 (1%)	0 (0%)	1 (4%)	0 (0%)	0 (0%)	0 (0%)
Hypothyroidism, n (%)	2 (2%)	2 (6%)	0 (0%)	0 (0%)	0 (0%)	0 (0%)
Osteoporosis, n (%)	5 (5%)	2 (6%)	1 (4%)	0 (0%)	2 (10%)	0 (0%)
Migraine, n (%)	1 (1%)	0 (0%)	0 (0%)	0 (0%)	0 (0%)	1 (10%)
Depression, n (%)	2 (2%)	0 (0%)	1 (4%)	0 (0%)	0 (0%)	1 (10%)
**Number of medications for comorbidities, n (%)**						
0	61 (62%)	21 (68%)	11 (44%)	9 (69%)	14 (70%)	6 (60%)
1	24 (24%)	9 (29%)	9 (36%)	1 (8%)	4 (20%)	1 (10%)
2	9 (9%)	1 (3%)	4 (16%)	1 (8%)	2 (10%)	1 (10%)
3	3 (3%)	0 (0%)	1 (4%)	1 (8%)	0 (0%)	1 (10%)
4	2 (2%)	0 (0%)	0 (0%)	1 (8%)	0 (0%)	1 (10%)
**Administration dose per day**						
NSAIDs [mg]	75 [10–180]	10 [10–10]	180 [75–180]	62.5 [75–110]	200 [200–200]	62.5 [12–450]
TA [tablets]	2 [1–4]	3 [2–4]	2 [1–3]	2 [2–4]	2 [1–4]	2 [1–3]
**Pain-NRS [points]**						
Baseline	6 [5–7]	7 [5–7]	5 [5–7]	6 [5–7]	5 [4–6]	6 [4–6]
After NSAIDs for 12 months	5 [4–6]	5 [4–7]	4 [4–6]	5 [3–7]	4 [3–5]	6 [4–6]
After TA for 12 months	4 [3–5]	5 [3–7]	4 [3–5]	4 [3–5]	3 [2–5]	4 [2–6]

*NRS, Numeric Rating Scale; NSAIDs, non-steroidal anti-inflammatory drugs; TA, tramadol hydrochloride/acetaminophen*.

*Data of sex, major diagnoses, and comorbidities are number and (%) of patients. Data of age, administration dose, and pain-NRS are medians and interquartile ranges [IQR]*.

**Table 2:** Course of pain-NRS.

	**Lumbago**	**Osteoarthritis**	**Rheumatoid arthritis**
Baseline	5 [5–6]	5 [6–7]	7 [7–7]
After NSAIDs for 12 months	4 [4–6]	3 [4–6]	5 [5–5]
After TA for 12 months	3 [4–5]	2 [3–4]	3 [4–5]

*NRS, Numeric Rating Scale; NSAIDs, non-steroidal anti-inflammatory drugs; TA, tramadol hydrochloride/acetaminophen*.

*Data of pain-NRS are medians and interquartile ranges [IQR]*.

The median baseline for eGFR was 84.0 ml/min/1.73 m^2^ (IQR, 67.6–102.0), the median baseline for AST was 20.0 U/L (IQR, 17.0–24.0), and the median baseline for ALT was 16.0 U/L (IQR, 11.0–22.0) (Table 3). eGFR level was significantly correlated with age at baseline (*r* = −0.606), after NSAID administration for 12 months (*r* = −0.682) and after TA administration for 12 months (*r* = −0.645).

**Table 3:** Course of laboratory levels.

	**Overall** **(*n* = 99)**	**Meloxicam** **(*n* = 31)**	**Loxoprofen** **(*n* = 25)**	**Diclofenac** **(*n* = 13)**	**Celecoxib** **(*n* = 20)**	**Other** **(*n* = 10)**
**eGFR [ml/min/1.73 m** ^**2**^ **]**						
Baseline	84.0 [67.6–102.0]	86.0 [75.7–104.0]	84.0 [65.1–93.8]	92.1 [65.0–116.5]	83.1 [57.1–98.1]	74.6 [64.3–88.3]
After NSAIDs for 12 months	72.8 [57.5–89.6]^*^	73.8 [60.9–89.6]^*^	72.1 [49.3–92.2]	72.6 [48.2–85.7]	76.2 [61.4–90.5]	66.7 [50.8–75.5]
After TA for 12 months	71.5 [57.7–88.7]^*^	72.9 [64.1–92.7]^*^	71.7 [53.7–90.4]	71.3 [56.2–97.5]	75.3 [58.0–84.5]	57.5 [43.8–77.3]
Changes during NSAIDs use	−13.8 [−25.0–0.0]	−18.8 [−28.7 to −5.9]^†^	−2.7 [−19.3–0.0]	−21.5 [−31.2 to −12.5]^†^	−1.8 [−14.1–0.0]	−14.8 [−27.6 to −5.8]
Changes during TA use	0.4 [−7.5–11.8]	4.0 [−7.5–14.0]	1.9 [−6.6–13.5]	1.5 [−1.1–15.0]	−2.8 [−9.9–11.0]	−8.5 [18.6–8.7]
**AST [U/L]**						
Baseline	20.0 [17.0–24.0]	20.0 [17.0–22.0]	22.0 [18.5–26.5]	21.0 [15.5–30.0]	19.0 [16.0–28.8]	22.5 [15.0–25.3]
After NSAIDs for 12 months	21.0 [16.0–25.0]	21.0 [16.0–24.0]	22.0 [16.5–26.0]	18.0 [15.5–21.0]	23.0 [15.0–31.0]	20.0 [16.5–22.5]
After TA for 12 months	19.0 [16.0–24.0]	19.0 [16.0–22.0]	22.0 [19.0–27.0]	18.0 [15.0–21.5]	19.5 [17.0–28.0]	17.0 [15.8–21.8]
Changes during NSAIDs use	0.0 [−12.5–17.6]	5.0 [−6.3–17.6]	−4.3 [−15.0–16.3]	−4.5 [−22.8–6.7]	1.6 [−16.9–18.5]	0.0 [−18.2–19.1]
Changes during TA use	−5.6 [−17.1–5.9]	−6.7 [−20.0–4.8]	0.0 [−7.7–14.4]	−5.3 [−14.8–0.0]	−10.2 [−16.6–6.5]	−16.7 [−20.0–3.3]
**ALT [U/L]**						
Baseline	16.0 [11.0–22.0]	15.0 [11.0–20.0]	17.0 [11.0–27.5]	15.0 [10.5–29.5]	17.5 [10.3–23.5]	16.5 [10.0–25.0]
After NSAIDs for 12 months	15.0 [10.0–21.0]	14.0 [10.0–19.0]	16.0 [9.0–28.5]	12.0 [9.0–24.5]	15.0 [11.0–25.0]	15.0 [10.5–20.3]
After TA for 12 months	14.0 [10.0–21.0]	12.0 [9.0–16.0]	17.0 [12.0–23.0]	12.0 [8.0–19.5]	14.5 [11.0–25.8]	13.5 [10.0–21.0]
Changes during NSAIDs use	0.0 [−25.0–23.5]	8.3 [−20.0–23.5]	−10.0 [−38.7–17.7]	−10.0 [−33.9−1.8]	6.5 [−29.6–37.2]	−9.2 [−25.3–19.8]
Changes during TA use	−9.1 [−27.6–8.3]	−11.1 [−40.0–7.7]	0.0 [−26.1–20.8]	−12.5 [−28.1–17.8]	−8.1 [−27.3–8.4]	−10.1 [−34.2–5.0]

*eGFR, estimated glomerular filtration rate; AST, aspartate transaminase; ALT, alanine transaminase; NSAIDs, non-steroidal anti-inflammatory drugs; TA, tramadol hydrochloride/acetaminophen*.

*Data are medians and interquartile ranges [IQR]. These data were analyzed using Kruskal–Wallis test and Steel–Dwass test. Significance level was set at <5%*.

* *Significant difference vs. baseline*.

† *Significant difference vs. celecoxib. eGFR after NSAIDs for 12 months and after TA for 12 months were significantly decreased than baseline, in overall and meloxicam*.

**Figure 2:**
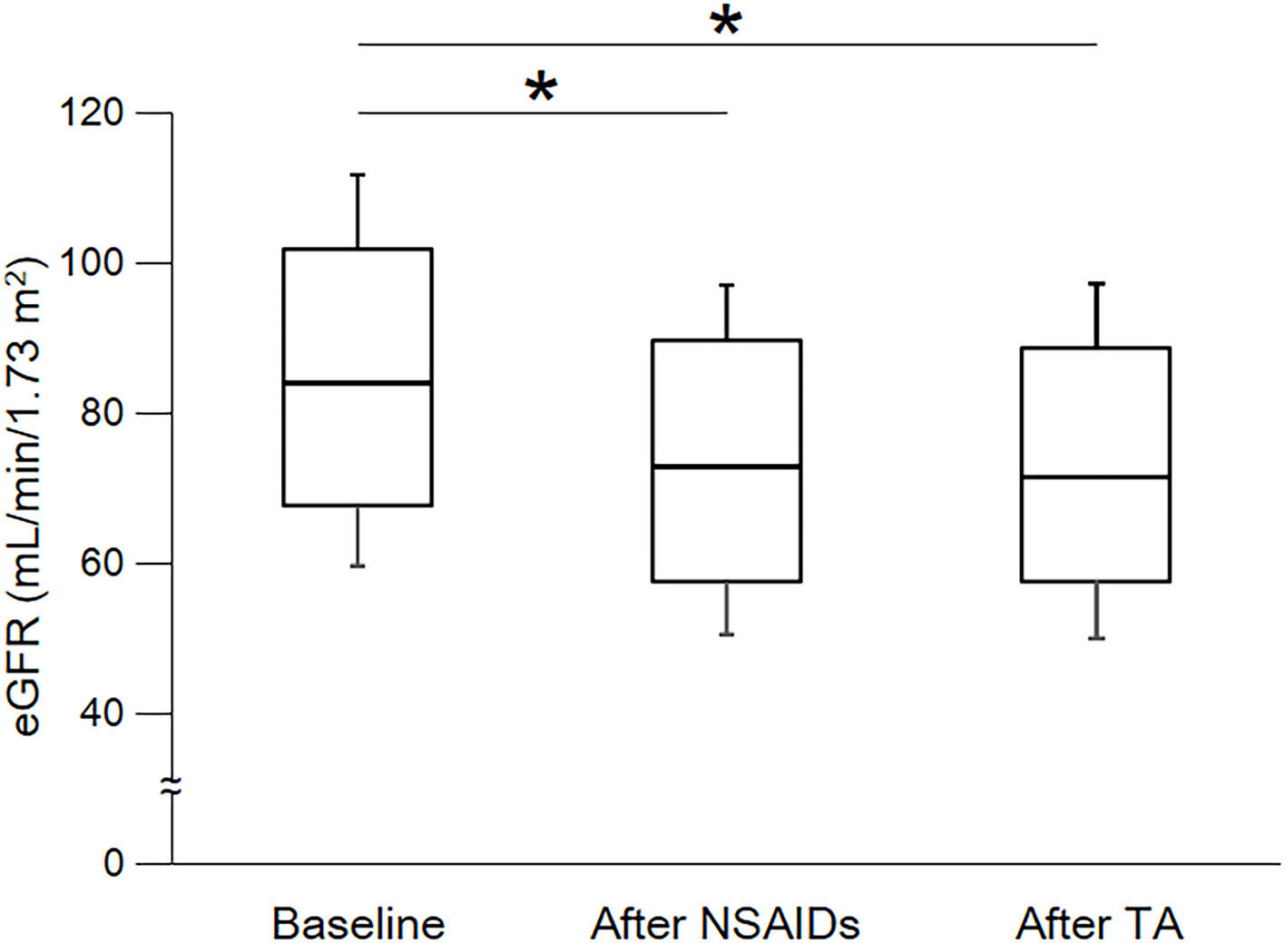
Course of eGFR. eGFR, estimated glomerular filtration rate; NSAIDs, non-steroidal anti-inflammatory drugs; TA, tramadol hydrochloride/acetaminophen. Each box plot represents the 75 percentile, median, and 25 percentile. Error bar shows standard deviation. eGFR after NSAIDs for 12 months and after TA for 12 months were significantly decreased than baseline. There was no significant difference between after NSAIDs for 12 months and after TA for 12 months. These data were analyzed using Friedman test and Steel–Dwass test. Significance level was set at <5%. ^*^Significant difference vs. baseline.

**Figure 3:**
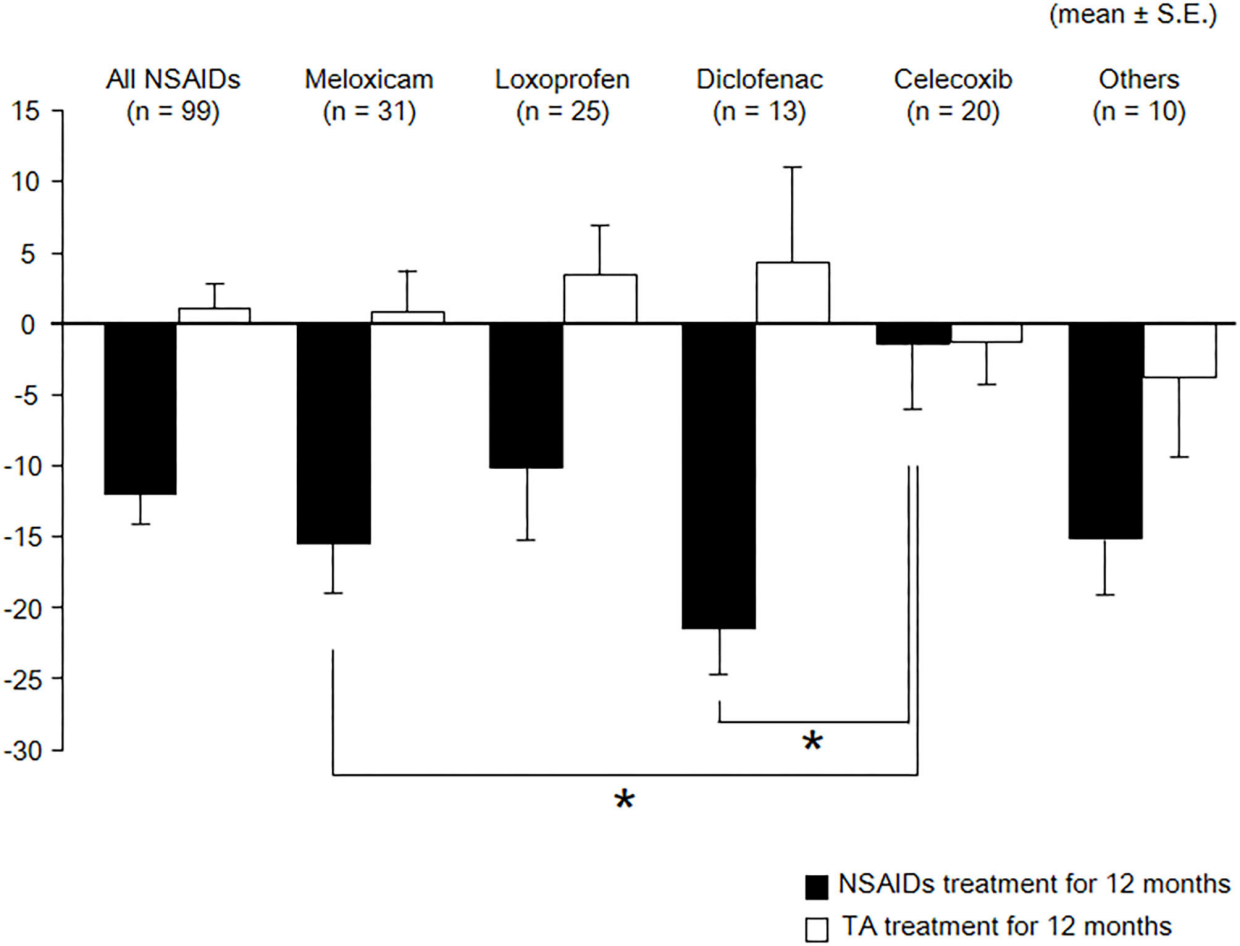
Course of eGFR among specific NSAIDs. eGFR, estimated glomerular filtration rate; TA, tramadol hydrochloride/acetaminophen. Values are means of change of eGFR, and the error bar shows standard error. Reduction of eGFR was significantly lesser in patients with celecoxib than those with meloxicam and diclofenac. These data were analyzed using Kruskal–Wallis test and Steel–Dwass test. Significance level was set at <5%. ^*^Significant difference among specific NSAIDs.

**Figure 4:**
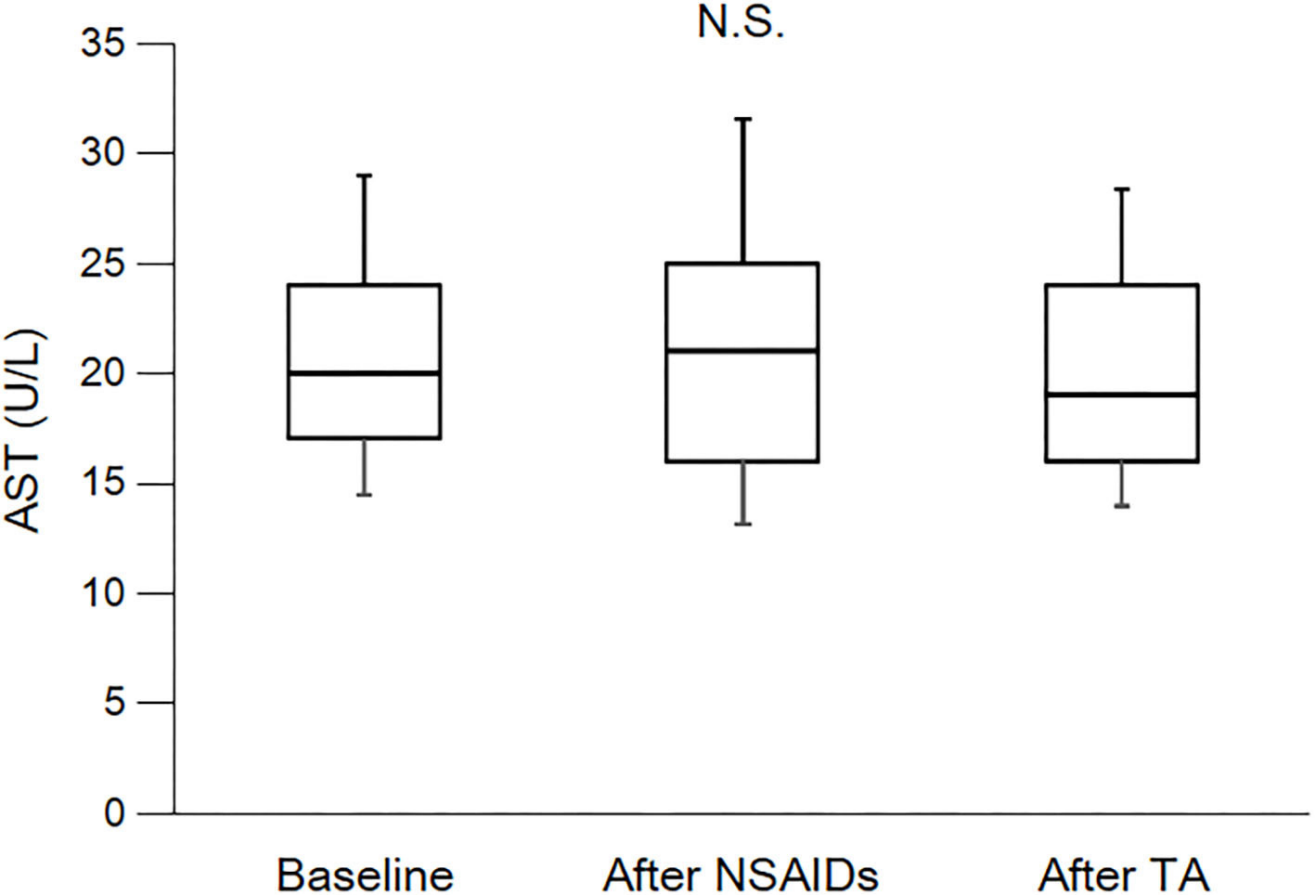
Course of AST. AST, aspartate transaminase; NSAIDs, non-steroidal anti-inflammatory drugs; TA, tramadol hydrochloride/acetaminophen. Each box plot represents the 75 percentile, median, and 25 percentile. Error bar shows standard deviation. There was no significant difference in each period. These data were analyzed using Friedman test and Steel–Dwass test. Significance level was set at <5%.

**Figure 5:**
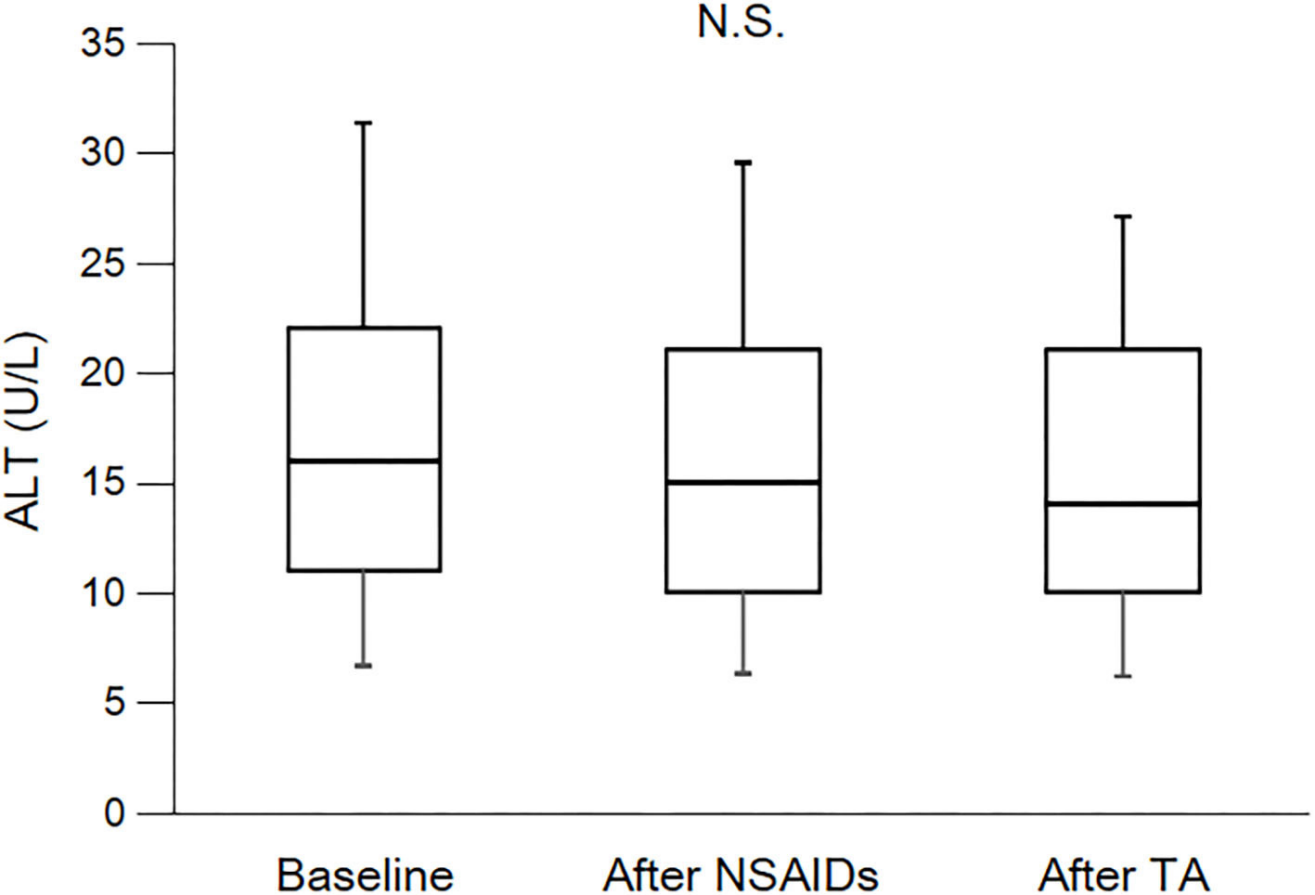
Course of ALT. ALT, alanine transaminase; NSAIDs, non-steroidal anti-inflammatory drugs; TA, tramadol hydrochloride/acetaminophen. Each box plot represents the 75 percentile, median, and 25 percentile. Error bar shows standard deviation. There was no significant difference in each period. These data were analyzed using Friedman test and Steel–Dwass test. Significance level was set at <5%.

As shown in Table 3 and Figure 2, eGFR levels after NSAID administration for 12 months followed by TA for 12 months were significantly reduced compared with baseline. eGFR was significantly reduced during the first 12 months with NSAID administration (median, from 84.0 to 72.8 ml/min/1.73 m^2^), whereas the reduction was not shown during the following 12 months with TA administration (median, 71.5 ml/min/1.73 m^2^). Some patients showed an increase of eGFR after cessation of NSAIDs and switching to TA. There was no significant difference in eGFR between after the 12-month NSAIDs period and after the 12-month TA period. With respect to the four specific NSAIDs, reduction of eGFR was significantly less in patients taking celecoxib (median, −1.8 ml/min/1.73 m^2^) than those on meloxicam or diclofenac (Figure 3). As shown in Table 3 and Figures 4, 5, there was no significant difference in AST or ALT in each period.

Table 4 shows the number of patients for each grade of the CKD category. Of the 99 patients, 37 patients (37%) experienced an increase in severity of at least one grade in the CKD category during the first 12 months with NSAID administration. Interestingly, the extent of severity varied by NSAID type, where 15% of patients on celecoxib (*n* = 3) were affected, compared with 77% of patients on diclofenac (*n* = 10) (*p* = 0.003). On the other hand, during the 24 months with NSAID and TA administration, 35 patients (35%) increased severity by at least one grade of the CKD category. There were 30 patients in more than three categories after NSAIDs for 12 months, whereas 28 patients in more than three categories after TA for 12 months. The number of patients increasing severity by at least one grade of the CKD category over 24 months showed no significant difference among the four specific NSAIDs used. The variables other than NSAID type were not significantly different between patients who fell into at least one grade worse of the CKD category or not (Table 5).

**Table 4:** Number of patients each grade of CKD category.

	**Overall** **(*n* = 99)**	**Meloxicam** **(*n* = 31)**	**Loxoprofen** **(*n* = 25)**	**Diclofenac** **(*n* = 13)**	**Celecoxib** **(*n* = 20)**	**Other** **(*n* = 10)**
**Baseline, n (%)**						
1	38 (38%)	13 (42%)	8 (32%)	8 (62%)	7 (35%)	2 (20%)
2	43 (43%)	16 (52%)	12 (48%)	2 (15%)	7 (35%)	6 (60%)
3a	13 (13%)	1 (3%)	3 (12%)	3 (23%)	5 (25%)	1 (10%)
3b	5 (5%)	1 (3%)	2 (8%)	0 (0%)	1 (5%)	1 (10%)
4	0 (0%)	0 (0%)	0 (0%)	0 (0%)	0 (0%)	0 (0%)
5	0 (0%)	0 (0%)	0 (0%)	0 (0%)	0 (0%)	0 (0%)
**After NSAIDs for 12 months, n (%)**						
1	23 (23%)	7 (23%)	8 (32%)	2 (15%)	5 (25%)	1 (10%)
2	46 (46%)	17 (55%)	7 (28%)	6 (46%)	11 (55%)	5 (50%)
3a	19 (19%)	5 (16%)	5 (20%)	3 (23%)	3 (15%)	3 (30%)
3b	9 (9%)	2 (6%)	4 (16%)	2 (15%)	1 (5%)	0 (0%)
4	2 (2%)	0 (0%)	1 (4%)	0 (0%)	0 (0%)	1 (10%)
5	0 (0%)	0 (0%)	0 (0%)	0 (0%)	0 (0%)	0 (0%)
**After TA for 12 months, n (%)**						
1	22 (22%)	8 (26%)	6 (24%)	3 (23%)	4 (20%)	1 (10%)
2	49 (49%)	18 (58%)	12 (48%)	5 (38%)	11 (55%)	3 (30%)
3a	16 (16%)	3 (10%)	2 (8%)	4 (31%)	4 (20%)	3 (30%)
3b	9 (9%)	1 (3%)	4 (16%)	0 (0%)	1 (5%)	3 (30%)
4	3 (3%)	1 (3%)	1 (4%)	1 (3%)	0 (0%)	0 (0%)
5	0 (0%)	0 (0%)	0 (0%)	0 (0%)	0 (0%)	0 (0%)
**Fell into at least worse one grade of CKD category, n (%)**						
during NSAIDs use (12 months)	37 (37%)	13 (42%)	7 (28%)	10 (77%)^†^	3 (15%)^*^	4 (40%)
during NSAIDs and TA use (24 months)	35 (35%)	11 (35%)	7 (28%)	8 (62%)	4 (20%)	5 (50%)

*NSAIDs, non-steroidal anti-inflammatory drugs; TA, tramadol hydrochloride/acetaminophen; CKD, chronic kidney disease*.

*Data are number and (%) of patients. Of 99 patients, 37 patients (37%) experienced an increase in severity of at least one grade in CKD category during first 12 months with NSAID administration. On the other hand, during 24 months with NSAIDs and TA administration, 35 patients (35%) increased severity by at least one grade of CKD category. These data were analyzed using chi-square test. Significance level was set at <5%*.

* *Significantly fewer number of patients*.

† *Significantly more number of patients*.

**Table 5:** Comparison between patients with fell into at least worse one grade of CKD category or not.

	**During NSAIDs use** **(12 months)**			**During NSAIDs and TA use** **(24 months)**	
	**Fell category** **(*n* = 37)**	**Not fell category** **(*n* = 62)**		**Fell category** **(*n* = 35)**	**Not fell category** **(*n* = 64)**
**Demographics**
Age [year]	76 [61–84]	72 [47–80]		73 [60–83]	73 [46–80]
Female, n (%)	11 (30%)	18 (29%)		12 (34%)	17 (27%)
**Major diagnoses (multiple allowed)**					
Lumbago, n (%)	21 (57%)	24 (39%)		18 (51%)	27 (42%)
Osteoarthritis, n (%)	7 (19%)	21 (34%)		9 (26%)	19 (30%)
Rheumatoid arthritis, n (%)	1 (3%)	2 (3%)		1 (3%)	2 (3%)
**Comorbidities**					
Diabetes, n (%)	3 (8%)	1 (2%)		2 (6%)	2 (3%)
Hypertension, n (%)	11 (30%)	11 (18%)		8 (23%)	14 (22%)
Chronic heart failure, n (%)	2 (5%)	1 (2%)		2 (6%)	1 (2%)
Dyslipidemia, n (%)	1 (3%)	0 (0%)		0 (0%)	1 (2%)
Hypothyroidism, n (%)	0 (0%)	2 (3%)		0 (0%)	2 (3%)
Osteoporosis, n (%)	1 (3%)	4 (6%)		1 (3%)	4 (6%)
Migraine, n (%)	0 (0%)	1 (2%)		0 (0%)	1 (2%)
Depression, n (%)	1 (3%)	1 (2%)		1 (3%)	1 (2%)
**Number of medications for comorbidities**					
0, n (%)	21 (57%)	40 (65%)		23 (66%)	38 (59%)
1, n (%)	9 (24%)	15 (24%)		6 (17%)	18 (28%)
2, n (%)	4 (11%)	5 (8%)		4 (11%)	5 (8%)
3, n (%)	1 (3%)	2 (3%)		1 (3%)	2 (3%)
4, n (%)	2 (5%)	0 (0%)		1 (3%)	1 (2%)
**Type of NSAIDs**					
Meloxicam, n (%)	13 (35%)	18 (29%)		11 (31%)	20 (31%)
Loxoprofen, n (%)	7 (19%)	18 (29%)		7 (20%)	18 (28%)
Diclofenac, n (%)	10 (27%)^†^	3 (5%)^*^		8 (23%)	5 (8%)
Celecoxib, n (%)	3 (8%)^*^	17 (27%)^†^		4 (11%)	16 (25%)
Other, n (%)	4 (11%)	6 (10%)		5 (14%)	5 (8%)
**Administration dose per day**					
NSAIDs [mg]	75 [10–150]	160 [10–200]		75 [10–180]	100 [10–180]
TA [tablets]	2 [1–4]	2 [1–3]		2 [1–4]	2 [1–4]

*NSAIDs, non-steroidal anti-inflammatory drugs; TA, tramadol hydrochloride/acetaminophen; CKD, chronic kidney disease*.

*Data of sex, major diagnoses, and comorbidities are number and (%) of patients. Data of age and administration doses are medians and interquartile ranges [IQR]. Of 99 patients, 37 patients (37%) experienced an increase in severity of at least one grade in CKD category during first 12 months with NSAID administration. On the other hand, during 24 months with NSAIDs and TA administration, 35 patients (35%) increased severity by at least one grade of CKD category. These data were analyzed using chi-square test or Mann–Whitney U-test. Significance level was set at <5%*.

* *Significantly fewer number of patients*.

† *Significantly more number of patients*.

## Discussion

The present study showed that NSAID administration for 12 months significantly reduced serum levels of eGFR. However, the reduction was not shown after 12 months of TA administration. Several patients showed an increase of eGFR upon cessation of NSAIDs followed by switching to TA.

Most forms of acute renal failure from NSAID administration are short-term and reversible upon NSAID discontinuation [8]. The adverse effects of NSAIDs are the consequences of inhibiting prostaglandin synthesis and can result in acute renal failure. Moreover, there is the possibility that chronic administration of any NSAIDs can cause chronic renal failure in some patients despite previous data suggesting it is safe [8, 19]. The underlying pathology of chronicity is considered chronic papillary necrosis or chronic interstitial nephritis [20]. NSAID administration for the short term for up to 6 weeks may preserve the chance for recovery [10]; however, there has previously been no study to test the reversibility of renal adverse effects after long-term NSAID use. The present study suggested that the eGFR was not reduced after the cessation of NSAIDs and switching to TA, but the reversibility as the change was not significant.

NSAIDs inhibit the peripheral production of prostaglandins and inflammatory processes [21]. NSAIDs could have a role in central neurons across the blood–brain barrier [22]. In osteoarthritis, NSAIDs could have favorable effects on articular cartilage and osteoarthritis progression, although there are no convincing data [23]. The present study showed a decrease in pain with a reduction of eGFR. The favorable and unfavorable effects of NSAIDs should be considered. Drug-induced renal failure is mostly induced by an antirheumatic drug, calcineurin inhibitors, an antitumor drug, and NSAIDs [24]. The nephrotoxic potential of dual or triple combinations of NSAIDs with renin–angiotensin system inhibitors and/or diuretics yields a high incidence of acute kidney injury [25, 26]. More than half of patients have no medications for comorbidities in the present study. NSAIDs also have serious adverse effects of heart attack and stroke. Other adverse effects include stomach pain, constipation, diarrhea, gas, heartburn, nausea, vomiting, and dizziness [27]. The patients in the present study showed no adverse effects. When the patients have any of the adverse effects, a physician should reconsider the subscription of NSAIDs.

The risk profiles of adverse effects are different for every NSAID [28–30]. A randomized control trial for patients with osteoarthritis or rheumatoid arthritis shows celecoxib treatment results in lower rates of renal adverse events than did ibuprofen [28]. In a meta-analysis of 114 clinical trials, Zhang et al. showed that rofecoxib intensified the risk for renal adverse effects. By contrast, among NSAIDs, celecoxib had a low risk for renal adverse effects [29]. Other NSAIDs were not significantly associated with the risk, although some trends were evident. Similarly, Winkelmayer et al. showed rofecoxib, ibuprofen, and indomethacin were associated with a higher risk of acute kidney injury than celecoxib [30]. In the present study, the reduction of renal function after administering NSAIDs for 12 months tended to be less in patients receiving celecoxib compared with patients receiving other NSAIDs.

TA combination tablets, which combine tramadol hydrochloride and acetaminophen, are a widely used analgesic [15]. Tramadol is a synthetic opioid receptor agonist with analgesic properties that also has a unique monoaminergic action through serotonin-noradrenaline reuptake inhibition [31]. Acetaminophen is one of the more traditional and better-tolerated among fast-acting analgesics that block pain through different pathways than opioids [32]. The effectiveness of TA in the treatment of chronic non-cancer pain is clinically acceptable, and improvements in pain contribute to improvements in quality of life in practice [15]. Most of the adverse effects of TA are non-serious [15, 33–35]; it is suggested that liver enzymes are elevated in the presence of acetaminophen at doses higher than normal therapeutic levels [36]. In addition, previous work showed that concomitant treatment with opioids does not lead to an elevation of liver enzyme levels [36]. Similarly, in our study, we did not observe any significant elevations in liver enzymes.

There are several limitations in the present study. First, the present study is a retrospective study limited only to patients receiving daily NSAIDs during the first 12 months followed by 12 months of administration of TA combination tablets daily. There is no group receiving only daily NSAIDs or TA combination tablets during the 24-month periods. The renal function might already have reached a stable but lower plateau in the present study. In addition, many patients had concomitant medications. Thus, our observations must be interpreted with caution. Second, the administration protocol was variable, and the overall impact of administration dose on serum levels was not determined. Third, patients were mostly of advanced age in the present study. The reduction of eGFR could be overestimated. Finally, we included only a small number of participants with different pain conditions at a single medical center. Further studies that investigate larger patient cohorts and additional treatment regimens are required to clarify the effects of long-term use of NSAIDs on serum levels.

## Conclusions

The present study suggests that patients who have undergone long-term NSAID therapy for 12 months can experience reversible or irreversible renal damage after the cessation of NSAIDs and switching to TA, as determined by measuring eGFR. Given this risk identified in our current series of patients, our data highlight the potential safety of utilizing multimodal analgesic therapies to minimize the chronic administration of NSAIDs wherever possible.

## Data Availability Statement

The raw data supporting the conclusions of this article will be made available by the authors, without undue reservation.

## Ethics Statement

The Research Ethics Committee of Amagasaki Central Hospital approved this study.

## Author Contributions

KM led the design of the study design with KH, HK, TI, and MY. KH led the analysis and the interpretation of data and drafted the manuscript with KM, HK, TI, and MY. All authors were involved in the interpretation of the results, writing of the manuscript, and they all approved the final manuscript.

## Conflict of Interest

KM has received payment from Merck, Pfizer, Eli Lilly, Ayumi, Mundi Pharma, Janssen, Nippon Zoki, and Daiichi Sankyo. The remaining authors declare that the research was conducted in the absence of any commercial or financial relationships that could be construed as a potential conflict of interest.

## References

[1] da Costa BR Reichenbach S Keller N Nartey L Wandel S Jüni P. Effectiveness of non-steroidal anti-inflammatory drugs for the treatment of pain in knee and hip osteoarthritis: a network meta-analysis. Lancet. (2017) 390:e21–33. 10.1016/S0140-6736(17)31744-0 28699595

[2] McAlindon TE Bannuru RR Sullivan MC Arden NK Berenbaum F Bierma-Zeinstra SM. OARSI guidelines for the non-surgical management of knee osteoarthritis. Osteoarthritis Cartilage. (2014) 22:363–88. 10.1016/j.joca.2014.01.003 24462672

[3] Wong JJ Côté P Sutton DA Randhawa K Yu H Varatharajan S. Clinical practice guidelines for the noninvasive management of low back pain: a systematic review by the Ontario Protocol for Traffic Injury Management (OPTIMa) Collaboration. Eur J Pain. (2017) 21:201–16. 10.1002/ejp.931 27712027

[4] Koes BW van Tulder M Lin CW Macedo LG McAuley J Maher C. An updated overview of clinical guidelines for the management of non-specific low back pain in primary care. Eur Spine J. (2010) 19:2075–94. 10.1007/s00586-010-1502-y 20602122 PMC2997201

[5] Schnitzer TJ. Update on guidelines for the treatment of chronic musculoskeletal pain. Clin Rheumatol. (2006) 25:S22–9. 10.1007/s10067-006-0203-8 16741783

[6] Qaseem A Wilt TJ McLean RM Forciea MA Clinical Guidelines Committee of the American College of Physicians. Non-invasive treatments for acute, subacute, and chronic low back pain: a clinical practice guideline from the American college of physicians. Ann Intern Med. (2017) 166:514–30. 10.7326/M16-2367 28192789

[7] Gooch K Culleton BF Manns BJ Zhang J Alfonso H Tonelli M. NSAID use and progression of chronic kidney disease. Am J Med. (2007) 120:e1–7. 10.1016/j.amjmed.2006.02.015 17349452

[8] Harirforoosh S Asghar W Jamali F. Adverse effects of nonsteroidal antiinflammatory drugs: an update of gastrointestinal, cardiovascular and renal complications. J Pharm Anti-Inflammat Sci. (2013) 16:821–47. 10.18433/J3VW2F 24393558

[9] Chou CI Shih CJ Chen YT Ou SM Yang CY Kuo SC. Adverse effects of oral nonselective and cyclooxygenase-2-selective NSAIDs on hospitalization for acute kidney injury: a nested case-control cohort study. Medicine. (2016) 95:e2645. 10.1097/MD.0000000000002645 26945352 PMC4782836

[10] Shukla A Rai MK Prasad N Agarwal V. Short-term non-steroid anti-inflammatory drug use in spondyloarthritis patients induces subclinical acute kidney injury: biomarkers study. Nephron. (2017) 135:277–86. 10.1159/000455167 28171854

[11] Gore M Sadosky A Leslie D Tai KS Seleznick M. Patterns of therapy switching, augmentation, and discontinuation after initiation of treatment with select medications in patients with osteoarthritis. Clin Ther. (2011) 33:1914–31. 10.1016/j.clinthera.2011.10.019 22088416

[12] Gore M Sadosky AB Leslie DL Tai KS Emery P. Therapy switching, augmentation, and discontinuation in patients with osteoarthritis and chronic low back pain. Pain Pract. (2012) 12:457–68. 10.1111/j.1533-2500.2011.00524.x 22230466

[13] Scholes D Stergachis A Penna PM Normand EH Hansten PD. Nonsteroidal antiinflammatory drug discontinuation in patients with osteoarthritis. J Rheumatol. (1995) 22:708–12. 7791168

[14] International Association for the Study of Pain (IASP) Subcommittee on Taxonomy. Classification of chronic pain. Descriptions of chronic pain syndromes and definitions of pain terms. Pain. (1986) 3:S1−226. 3461421

[15] Yoshizawa K Kawai K Fujie M Suzuki J Ogawa Y Yajima T. Overall safety profile and effectiveness of tramadol hydrochloride/acetaminophen in patients with chronic noncancer pain in Japanese real-world practice. Curr Med Res Opin. (2015) 31:2119–29. 10.1185/03007995.2015.1091975 26359328

[16] Matsuo S Imai E Horio M Yasuda Y Tomita K Nitta K. Revised equations for estimated GFR from serum creatinine in Japan. Am J Kidney Dis. (2009) 53:982–92. 10.1053/j.ajkd.2008.12.034 19339088

[17] Stevens PE Levin A Kidney Disease: Improving Global Outcomes Chronic Kidney Disease Guideline Development Work Group Members. Evaluation and management of chronic kidney disease: synopsis of the kidney disease: improving global outcomes 2012 clinical practice guideline. Ann Intern Med. (2013) 158:825–30. 10.7326/0003-4819-158-11-201306040-00007 23732715

[18] Farrar JT Young JP Jr LaMoreaux L Werth JL Poole MR. Clinical importance of changes in chronic pain intensity measured on an 11-point numerical pain rating scale. Pain. (2001) 94:149–58. 10.1016/S0304-3959(01)00349-9 11690728

[19] Nderitu P Doos L Jones PW Davies SJ Kadam UT. Non-steroidal anti-inflammatory drugs and chronic kidney disease progression: a systematic review. Fam Prac. (2013) 30:247–55. 10.1093/fampra/cms086 23302818

[20] Kleinknecht D. Interstitial nephritis, the nephritic syndrome and chronic renal failure secondary to non-steroidal anti-inflammatory drugs. Semin Nephrol. (1995) 15:228–35. 7631049

[21] Malfait AM Schnitzer TJ. Towards a mechanism-based approach to pain management in osteoarthritis. Nat Rev Rheumatol. (2013) 9:654–64. 10.1038/nrrheum.2013.138 24045707 PMC4151882

[22] Vardeh D Wang D Costigan M Lazarus M Saper CB Woolf CJ. COX2 in CNS neural cells mediates mechanical inflammatory pain hypersensitivity in mice. J Clin Invest. (2009) 119:287–94. 10.1172/JCI37098 19127021 PMC2631301

[23] Ding C. Do NSAIDs affect the progression of osteoarthritis? Inflammation. (2002) 26:139–42. 10.1023/A:1015504632021 12083420

[24] Usui J Yamagata K Imai E Okuyama H Kajiyama H Kanamori H. Clinical practice guideline for drug-induced kidney injury in Japan 2016: digest version. Clin Exp Nephrol. (2016) 20:827–31. 10.1007/s10157-016-1334-0 27714545 PMC5127866

[25] Lapi F Azoulay L Yin H Nessim SJ Suissa S. Concurrent use of diuretics, angiotensin converting enzyme inhibitors, and angiotensin receptor blockers with non-steroidal anti-inflammatory drugs and risk of acute kidney injury: nested case-control study. BMJ. (2013) 346:e8525. 10.1136/bmj.e8525 23299844 PMC3541472

[26] Dreischulte T Morales DR Bell S Guthrie B. Combined use of nonsteroidal anti-inflammatory drugs with diuretics and/or renin-angiotensin system inhibitors in the community increases the risk of acute kidney injury. Kidney Int. (2015) 88:396–403. 10.1038/ki.2015.101 25874600

[27] Food US Drug Administration. Medication Guide for Non-Steroidal Anti-Inflammatory Drugs (NSAIDs). (2021). Available online at: https://www.fda.gov/media/73092/download (accessed, March 27, 2021).

[28] Nissen SE Yeomans ND Solomon DH Lüscher TF Libby P Husni ME. Cardiovascular safety of celecoxib, naproxen, or ibuprofen for arthritis. N Engl J Med. (2016) 375:2519–29. 10.1056/NEJMoa1611593 27959716

[29] Zhang J Ding EL Song Y. Adverse effects of cyclooxygenase 2 inhibitors on renal and arrhythmia events: meta-analysis of randomized trials. JAMA. (2006) 296:1619–32. 10.1001/jama.296.13.jrv60015 16968832

[30] Winkelmayer WC Waikar SS Mogun H Solomon DH. Nonselective and cyclooxygenase-2-selective NSAIDs and acute kidney injury. Am J Med. (2008) 121:1092–8. 10.1016/j.amjmed.2008.06.035 19028206

[31] Raffa RB Friderichs E Reimann W Shank RP Codd EE Vahght JL. Opioid and non-opioid components independently contribute to the mechanism of action of tramadol, an ‘atypical' opioid analgesic. J Pharmacol Exp Ther. (1992) 260:275–85. 1309873

[32] Józwiak-Bebenista M Nowak JZ. Paracetamol: mechanism of action, applications and safety concern. Acta Pol Pharm. (2014) 71:11–23. 24779190

[33] Inoue Y Nishimura A Taguma K. A long-term (52-week) study of tramadol hydrochloride/acetaminophen combination tablet for chronic pain. J Bone Joint Surg. (2012) 31:88–97.

[34] Mejjad O Serrie A Ganry H. Epidemiological data, efficacy and safety of a paracetamol–tramadol fixed combination in the treatment of moderate-tosevere pain. SALZA: a post-marketing study in general practice. Curr Med Res Opin. (2011) 27:1013–20. 10.1185/03007995.2011.565045 21401445

[35] Moore RA McQuay HJ. Prevalence of opioids adverse events in chronic nonmalignant pain: systematic review of randomized trial of oral opioids. Arthritis Res Ther. (2005) 7:1046–51. 10.1186/ar1526 PMC1257433 16207320

[36] Watkins PB Kaplowitz N Slattery JT Colonese CR Colucci SV Stewart PW. Aminotransferase elevations in healthy adults receiving 4 grams of acetaminophen daily: a randomized controlled trial. JAMA. (2006) 296:87–93. 10.1001/jama.296.1.87 16820551

